# Genetic characterisation of *PPARG*, *CEBPA* and *RXRA*, and their influence on meat quality traits in cattle

**DOI:** 10.1186/s40781-016-0095-3

**Published:** 2016-04-01

**Authors:** Daniel Estanislao Goszczynski, Juliana Papaleo Mazzucco, María Verónica Ripoli, Edgardo Leopoldo Villarreal, Andrés Rogberg-Muñoz, Carlos Alberto Mezzadra, Lilia Magdalena Melucci, Guillermo Giovambattista

**Affiliations:** Instituto de Genética Veterinaria “Ing. Fernando Noel Dulout” (IGEVET), CONICET, Facultad de Ciencias Veterinarias, Universidad Nacional de La Plata, CC 296, La Plata, B1900AVW Argentina; Unidad Integrada INTA Balcarce-Facultad de Ciencias Agrarias, Universidad Nacional de Mar del Plata, Balcarce, Argentina; Fellow of the Consejo Nacional de Investigaciones Científicas y Técnicas (CONICET), Buenos Aires, Argentina

**Keywords:** Polymorphism, Variation, Association, Cows, Beef, SNPs

## Abstract

**Background:**

Peroxisome proliferator-activated receptor gamma (PPARG), CCAAT/enhancer binding protein alpha (CEBPA) and retinoid X receptor alpha (RXRA) are nuclear transcription factors that play important roles in regulation of adipogenesis and fat deposition. The objectives of this study were to characterise the variability of these three candidate genes in a mixed sample panel composed of several cattle breeds with different meat quality, validate single nucleotide polymorphisms (SNPs) in a local crossbred population (Angus - Hereford - Limousin) and evaluate their effects on meat quality traits (backfat thickness, intramuscular fat content and fatty acid composition), supporting the association tests with bioinformatic predictive studies.

**Results:**

Globally, nine SNPs were detected in the *PPARG* and *CEBPA* genes within our mixed panel, including a novel SNP in the latter. Three of these nine, along with seven other SNPs selected from the Single Nucleotide Polymorphism database (SNPdb), including SNPs in the *RXRA* gene, were validated in the crossbred population (*N* = 260). After validation, five of these SNPs were evaluated for genotype effects on fatty acid content and composition. Significant effects were observed on backfat thickness and different fatty acid contents (*P* < 0.05). Some of these SNPs caused slight differences in mRNA structure stability and/or putative binding sites for proteins.

**Conclusions:**

*PPARG* and *CEBPA* showed low to moderate variability in our sample panel. Variations in these genes, along with *RXRA*, may explain part of the genetic variation in fat content and composition. Our results may contribute to knowledge about genetic variation in meat quality traits in cattle and should be evaluated in larger independent populations.

**Electronic supplementary material:**

The online version of this article (doi:10.1186/s40781-016-0095-3) contains supplementary material, which is available to authorized users.

## Background

Fat content and composition are considered major economically important traits in livestock, since variations in these two factors affect several meat properties [1]. These traits are the result of several biological processes, such as adipogenesis, lipolysis and fatty acid-transfer. Therefore, a part of the variability produced by these processes may be attributed to the genetic variants of the pathway members. Peroxisome proliferator-activated receptor gamma (PPARG), CCAAT/enhancer binding protein alpha (CEBPA) and retinoid X receptor alpha (RXRA) are important nuclear transcription factors involved in numerous cellular processes [2], and are considered key molecules in regulation of adipogenesis. PPARG and CEBPA are induced early in the signaling pathway, they work together to trigger the process and regulate each other [3]. PPARG acts as heterodimer with RXRA, which belongs to a family of nuclear receptors that act as homodimers and heterodimers. In view of their roles, the genetic control of adipogenesis by *PPARG*, *CEBPA* and *RXRA* may be important and helpful for animal improvement.

During the last years, SNPs in *PPARG* and *CEBPA* have been associated with a group of meat quality traits in Chinese and Korean cattle, including tenderness, backfat thickness, water holding capacity, fatty acid composition, weight at slaughter and marbling, among others [4–9]. However, those works have been performed almost entirely using Asian cattle under feedlot conditions and the results might not be necessarily comparable with researches performed with other breeds under pasture-based feeding. For instance, these two conditions may activate specific metabolic pathways governed by different genes. Nowadays, most of the exported beef in the world is produced on pasture-based systems [10], as in countries like Argentina, Brazil, New Zealand, Paraguay and Uruguay, among others.

In this context, we searched for gene variants in *PPARG* and *CEBPA* in a sample set composed of nine cattle breeds with different meat quality. Then, we validated some of these SNPs, along with SNPs in the *RXRA* gene, in a local Angus-Hereford-Limousin crossbred population (*N* = 260) fed on pasture-based conditions. We used this population to evaluate the association of the SNPs with intramuscular fat content (IF), backfat thickness (BT) and fatty acid composition. Finally, we analysed the molecular effects of these SNPs through bioinformatic predictive tools.

## Methods

### Animal samples and DNA extraction

Two groups of samples were collected: the first group comprised blood samples from 43 unrelated purebred animals (Angus, *n* = 5; Brahman, *n* = 5; Creole, *n* = 5; Hereford, *n* = 5; Holstein, *n* = 5; Limousin, *n* = 4; Nellore, *n* = 4; Shorthorn, *n* = 5; Wagyu, *n* = 5), which were used to identify polymorphisms in the bovine *PPARG* and *CEBPA* genes. The second group comprised 260 steers (15–29 month-old), born between 2006 and 2010, which were used to perform population analyses, including SNP validation and association tests. This group of animals had been used in previous studies to evaluate crossbreeding systems under pasture grazing with strategic supplementation at the Experimental Station of the National Institute of Agricultural Technology (INTA, Balcarce, Argentina; Coordinates: 37°49S 58°15 W). Steers included: purebred Angus -A- (*n* = 44) and Hereford -H- (*n* = 26) steers, their crossbreeds F1 and F2 -½AH- (*n* = 95), reciprocal backcrosses -¾A and ¾ H- (*n* = 54), and steers produced by mating Limousin -L- sires with F1 crossbred cows -LX- (*n* = 41) (Additional file 1: Table S1). Fifty-four sires were used, including 17 A sires (1-16 steers), 18 H sires (1-11 steers), 8 AH sires (1-7 steers), 8 HA sires (1-7 steers) and 4 L sires (1-34 steers). L sires were mated only with AH and HA cows and, every year, some of the A and H sires were mated with more than one genetic group.

All animals grazed a sown pasture (predominantly *Lolium multiflorum*, *Dactylis glomerata*, *Bromus catarthicus*, *Trifolium repens* and *Trifolium pratense*). They were slaughtered in eight groups and meat blocks were taken from the 13th rib to perform association studies (Additional file 2: Table S1). The decision to sample this experimental population instead of other commercial cattle populations was based on the availability of reliable information in terms of phenotypic data, management and genetic background of the animals.

DNA was isolated from blood lymphocytes using Wizard® Genomic DNA purification kit (Promega, Madison, WI, USA) following the instructions of the supplier, and from meat samples as previously described in [11].

### Re-sequencing study of the bovine *PPARG* and *CEBPA* genes

To amplify the coding regions of the *PPARG* and *CEBPA* genes, ten pairs of primers were designed according to the DNA sequences available in GenBank [Gene IDs: 281677, 281993] (Additional file 2: Table S2). PCR reactions were performed, verified, purified and sequenced as described in [12]. Sequences were aligned using CLUSTAL-X 2.1 [13] and variants were defined by direct comparison with the bovine reference sequences.

### SNP selection and genotyping

As the crossbred population used to validate SNPs and perform association tests included animals from Angus, Hereford and Limousin (pure or crossbred), only some of the SNPs detected in the re-sequencing stage were considered for further validation. In other words, many of the SNPs detected by re-sequencing showed no variation in the Taurine breeds and were not considered, since they would probably show no variation in the crossbred population. For this reason, additional SNPs were selected from dbSNP [14] to have a better covering of the length of the genes using markers with proven variation in Taurine breeds. At this stage, the addition of another candidate gene was decided, and SNPs in the *RXRA* gene were also selected from dbSNP to be validated and tested for associations. Genotyping was performed by the Neogen genotyping service (USA) using the Sequenom platform [15].

### Meat quality measurement

Fatty acid content and composition measurements were gathered from blocks of meat obtained from the 260 animals of the crossbred population to perform association studies. These blocks, corresponding to the *Longissimus dorsi* muscle (13th rib) were extracted from the carcass 24 h after slaughter. Backfat Thickness (BT) was measured perpendicular to the outer surface at a point three quarters of the length of the *Longissimus dorsi* muscle from the end of the loin bone, and expressed in millimetres. The Intramuscular Fat (IF) and fatty acid composition were then measured as described in [12]. The measured fatty acids were: myristic acid (C14:0); myristoleic acid (C14:1); palmitic acid (C16:0); palmitoleic acid (C16:1); stearic acid (C18:0); oleic acid (C18:1 cis-9); linoleic acid (C18:2 cis-9,12); γ-linolenic acid (C18:3 cis-6,9,12); α-linolenic acid (C18:3 cis-9,12,15); total saturated fatty acids (SFA); total monounsaturated fatty acids (MUFA); and proportion between omega-6 and omega-3 fatty acids (Ω6/Ω3). The fatty acid contents were expressed as percentage of total fatty acids per sample. C20:0 and other long-chain fatty acids were not included in the analysis since their percentages were lower than 0.5 %. The means, standard deviations, minimum and maximum values of all these measurements were included in Additional file 3: Table S3.

### Statistical Analysis and association with meat quality

Haplotypes and linkage disequilibrium (LD) among SNPs were estimated and visualized on HAPLOVIEW v4.2 [16] using the four gamete rule and the solid spine of LD. Allele frequencies and Hardy-Weinberg equilibrium (HWE) were analysed using GENEPOP software [17]. The 95 % confidence intervals for allele frequencies were computed using the binomial distribution implemented in R with the binom.confint function (http://cran.r-project.org/web/packages/binom/). Values for unbiased expected (h_e_) and observed (h_o_) heterozygosity were calculated using ARLEQUIN v3.5 [18].

The association of genotypes with BT, IF and fatty acid composition was evaluated using mixed models. BT and IF were analysed according to the following model:

$$ {Y}_{ijkl} = \mu + G{G}_j + S{G}_k + a\ Z{1}_i + d\ Z{2}_i + \beta\ {W}_i + {S}_l + {e}_{ijkl} $$

Where *Y*_*ijkl*_ = observed value of the phenotypic variable, *μ* = intercept, *GG*_*j*_ = fixed effect of the j^th^ genetic group, *SG*_*k*_ = fixed effect of the k^th^ slaughter group, *a* = additive effect for the SNP, *Z1*_*i*_ is the incidence variable for the additive effect (that is 0 for one of the homozygous genotypes, 1 for the heterozygous genotype and 2 for the alternative homozygous one), *d* = dominant effect for the SNP, *Z2*_*i*_ is the incidence variable for the dominance effect (that is 0 for both of the homozygous genotypes and 1 for the heterozygous genotype), *βW*_*i*_ = animal weight covariate for the i^th^ animal, *S*_*l*_ = random effect of the l^th^ sire, *e*_*ijkl*_ = random error. The same single trait model was used for fatty acid composition variables, but using ether extract instead of animal weight as covariate.

All statistical analyses were performed using the MIXED procedure of SAS software [19]. When the additive or dominance effects of the SNP were statistically significant (*P* < 0.05), the substitution effect (*α*) was calculated considering the frequencies of the major and minor alleles (*p* and *q*, respectively) and using the following equation [20]:

$$ \alpha =a+d\left(q-p\right) $$

The variance explained by the SNP (*σ*_*SNP*_^2^) was also estimated for each SNP-trait test as follows:

$$ {\sigma}_{SNP}^2=100\kern0.75em \times \frac{\left(RMS-FMS\right)}{RMS} $$

where RMS is the residual of the reduced model (SNP effect excluded), and FMS is the residual of the full model (SNP effect included). After all trait-SNP tests were performed, the false discovery rate (FDR) for multiple comparisons was controlled through the Benjamini & Hochberg method [21].

### Bioinformatic analyses

The SNPs were also analysed through different bioinformatic prediction tools. For synonymous SNPs, changes in codon frequency usage were analysed through the Codon Usage Database [22]. For SNPs located in 5’ UTR regions, the complete 5’ UTR fragments of the RNA sequences were run on the Mfold Web Server [23] to compare stability among variants. These fragments were also analysed using RBPDB [24], the database of RNA-binding protein (RBP) specificities, considering a threshold of 0.8 to identify putative RBP binding sites. Finally, variations located in promoter regions were analysed through PhysBinder [25], considering all human and murine models available and the “average” threshold to predict putative transcription factor binding sites.

## Results and discussion

### Re-sequencing study

A total of seven SNPs were identified in the *PPARG* gene using the panel of nine breeds. All of them had been previously reported and most showed very low frequencies. In fact, no homozygous genotypes were detected for any of the alternative alleles. Three of the SNPs were located in UTR regions: rs207671117 (5’ UTR), rs211388309 (3’ UTR) and rs207724742 (3’ UTR); the first in Angus and Hereford, and the other two in Brahman and Nellore. The remaining four SNPs (rs207739706, rs41610552, rs110194439 and rs42661651) were detected in non-coding regions in different breeds (Table 1).

**Table 1:** Genetic variants detected in the bovine *PPARG* and *CEBPA* genes*.* Variants were identified by re-sequencing a mixed sample panel (*N* = 43) composed of cattle breeds with different meat quality (Angus, Brahman, Creole, Hereford, Holstein, Limousin, Nellore, Shorthorn, Wagyu)

Gene	Reference number	Region	AA breeds	AB breeds	BB breeds
*PPARG*	rs207671117	Exon 1 - 5’ UTR	Brahman, Creole, Holstein, Limousin, Nellore, Shorthorn, Wagyu	Angus, Hereford	-
	rs207739706	Intron 1	Angus, Creole, Hereford, Holstein, Wagyu	Brahman	-
	rs41610552	Intron 2	-	Angus, Brahman, Creole, Hereford, Holstein	-
	rs211388309	Exon 7 - 3’ UTR	Angus, Creole, Hereford, Holstein, Nellore, Wagyu	Brahman	-
	rs207724742	Exon 7 - 3’ UTR	Angus, Creole, Hereford, Holstein, Wagyu	Brahman, Nellore	-
	rs110194439	Downstream	Angus, Brahman, Creole, Hereford, Holstein, Nellore	Wagyu	-
	rs42661651	Downstream	Creole, Holstein, Nellore	Angus, Brahman, Hereford, Wagyu	-
*CEBPA*	ss1751108604	Exon	Angus, Creole, Hereford, Holstein, Limousin, Shorthorn	Brahman, Nellore, Wagyu	-
	rs110793792	Exon	Nellore	Angus, Brahman, Creole, Hereford, Holstein, Limousin, Wagyu	Shorthorn

**Fig. 1:**
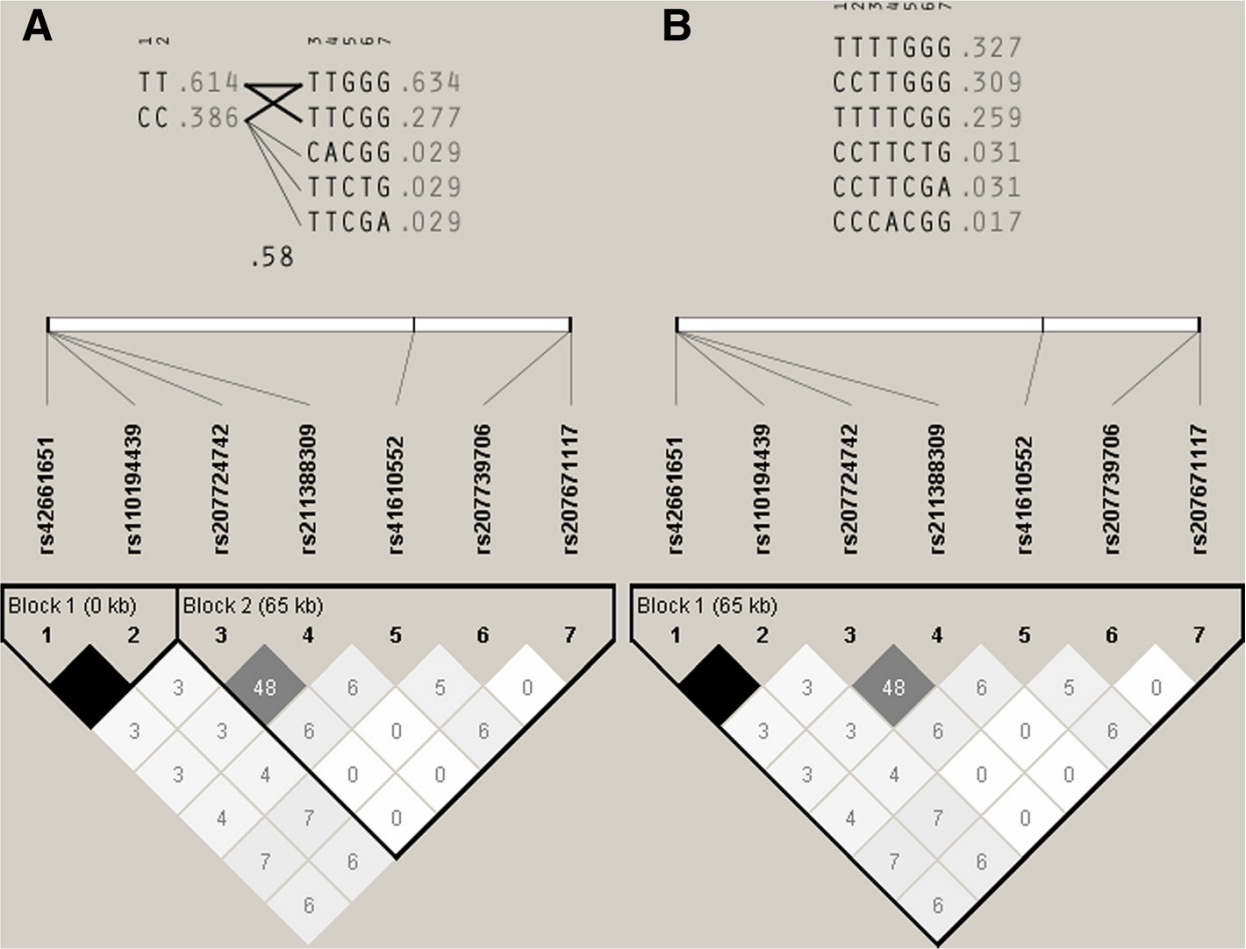
Haplotypes (upper part) and linkage disequilibrium (lower part) among SNPs in the *PPARG* gene estimated in a mixed sample panel (*n* = 43). Blocks were estimated using the four gamete rule (**a**) and solid spine of LD (**b**). In both cases, r2 values are indicated inside the boxes and blocks are indicated in thick lines

The haplotype and LD analysis, estimated with the four gamete rule, showed two blocks: a small one (3’ end), composed by two completely linked SNPs (rs42661651 and rs110194439), and a big one that included the other five SNPs and consisted of five haplotypes, with three of them in very low frequencies (Fig. 1). The same study, but estimated with solid spine of LD, showed one big block constituted by all the SNPs, with three haplotypes in very low frequencies.

Only two SNPs were detected in the *CEBPA* gene. These SNPs were located in the coding region of the gene, which comprised only one exon, and one of them had no previous reports in dbSNP. This novel SNP (ss1751108604) was detected in both Zebuine breeds (Brahman and Nellore) and the Japanese breed Wagyu. This SNP caused an amino acid change involving two neutral and polar residues (Ser139Asn). The other one was a synonymous SNP (rs110793792), widely distributed among the breeds, with the exception of Nellore and Shorthorn, which showed different homozygous genotypes for the mutation and no variability within the samples (Table 1).

Nine mutations were detected in total in this first stage. In other terms, we detected one SNP every 423 bp over 3809 bp analysed. Considering subspecies distribution, our variation values translate to one SNP every 762 bp for *Bos taurus* and one SNP every 544 bp for *B. indicus*. As expected, variability was higher in the Zebuine group. A few years ago, the Bovine HapMap Consortium [26] obtained one SNP every 714 bp for Angus or Holstein, and one SNP every 285 bp for Brahman. Therefore, the variability obtained in this work was similar in the case of Taurine breeds but lower for Zebuine breeds. On the other hand, the variability observed here was lower than that reported lately by our group for the *LIPE* gene in this same sample panel*,* where a SNP was detected every 123 bp [12]. This is consistent with the roles these genes play in lipid metabolism, since *PPARG* and *CEBPA* are key regulators in the first stages of fat deposition, among other processes, and *LIPE* codifies for an enzyme with very specific functions in lipid hydrolysis. In this scenario, *LIPE* may be subject to a less selective pressure than *PPARG* and *CEBPA*. In the case of *PPARG*, mutations were generally detected in low frequencies, which generated large linkage blocks with few haplotypes carrying most of the variation.

### Allelic and genotypic frequencies

A group of SNPs was selected either from the results of the re-sequencing study or dbSNP to perform validation. In the case of *PPARG*, two SNPs were selected from the re-sequencing study (rs207671117 and rs41610552) and two other SNPs were selected from dbSNP. These two SNPs from the dbSNP had been previously associated with a group of meat quality traits: rs42016945, located upstream of PPARG-2 and also part of the 5’ UTR of splice-variant 1 (PPARG-1), and rs109613657, which caused an amino acid change in the seventh exon (Glu448His). For *CEBPA*, we selected rs110793792 from the re-sequencing stage, since the novel SNP (ss1751108604) showed no variability in the Taurine breeds despite its novelty, and rs210446561 from the dbSNP. We also selected four SNPs from the *RXRA* gene. Since this gene was included in the study after the re-sequencing stage and there were no previous reports of associations with meat quality traits to our knowledge, the SNPs were chosen directly from dbSNP. These SNPs were rs209839910 (Pro108Ser), located in the second exon, rs136289117 (synonymous), located in the ninth exon, and rs133517803 and rs207774429, which may be located in the first intron or a putative promoter region for the splice-variant 3.

Regarding the SNPs in *PPARG*, rs207671117 showed low variability in subpopulations A, ¾ H, ½AH and LX (Minimum Allele Frequency [MAF] ≥ 0.02), and showed no variability in H and ¾A. On the other hand, SNPs rs41610552 and rs42016945 showed moderate allele frequencies among the subpopulations (MAF ≥ 0.12). Surprisingly, SNP rs109613657 showed no variability at all (Table 2). The HWE test showed no significant deviations from the theoretical proportions, with the exception of rs42016945 in subpopulation LX (*P* < 0.05) (Table 3). The unbiased expected heterozygosity (h_e_) of the two balanced SNPs (rs41610552 and rs42016945) varied between 0.22 (¾ H) and 0.49 (LX). Observed heterozygosity (h_o_) varied between 0.25 (¾ H) and 0.55 (LX). When linkage disequilibrium was analysed, rs207671117 and rs42016945 showed a small block with three haplotypes, two of them with more than 95 % of the haplotype frequencies (Additional file 4: Figure S1).

**Table 2:** Observed allele frequencies and 95 % confidence intervals for SNPs in the *PPARG*, *CEBPA* and *RXRA* genes in an Argentinean crossbred population (Angus-Hereford-Limousin). A: purebred Angus; H. purebred Hereford; ¾A: 75 % Angus steers; ¾ H: 75 % Hereford steers; ½AH: 50 % Angus -50 % Hereford steers; LX: Limousine crossbred steers. N: number of animals genotyped efficiently for that SNP per genetic group. SNP rs109613657 (*PPARG*) showed no variability

	Population						
N	44	26	30	24	95	41	260
rs41610552 (*PPARG*)	A	H	¾A	¾ H	½AH	LX	Global
C	68.18 (57.39 - 77.71)	82.00 (68.56 - 91.42)	76.67 (63.96 - 86.62)	87.50 (74.75 - 95.27)	76.84 (70.19 - 82.64)	60.00 (48.44 - 70.80)	74.22 (70.22 - 77.95)
G	31.82 (22.29 - 42.61)	18.00 (8.58 - 31.44)	23.33 (13.38 - 36.04)	12.50 (4.73 - 25.25)	23.16 (17.36 - 29.81)	40.00 (29.20 - 51.56)	25.78 (22.05 - 29.78)
rs207671117 (*PPARG*)	A	H	¾A	¾ H	½AH	LX	Global
A	4.55 (1.25 - 11.23)	0.00 (0.00 - 6.85)	0.00 (0.00 - 5.96)	2.08 (0.05 - 11.07)	3.16 (1.17 - 6.75)	6.10 (2.01 - 13.66)	3.08 (1.77 - 4.95)
G	95.45 (88.77 - 98.75)	100.00 (93.15 - 100.00)	100.00 (94.04 - 100.00)	97.92 (88.93 - 99.95)	96.84 (93.25 - 98.83)	93.90 (86.34 - 97.99)	96.92 (95.05 - 98.23)
rs42016945 (*PPARG*)	A	H	¾A	¾ H	½AH	LX	Global
C	68.18 (57.39 - 77.71)	78.85 (65.30 - 88.94)	68.33 (55.04 - 79.74)	81.25 (67.37 - 91.05)	75.79 (69.06 - 81.70)	65.85 (54.55 - 75.97)	72.88 (68.84 - 76.66)
T	31.82 (22.29 - 42.61)	21.15 (11.06 - 34.70)	31.67 (20.26 - 44.96)	18.75 (8.95 - 32.63)	24.21 (18.30 - 30.94)	34.15 (24.03 - 45.45)	27.11 (23.34 - 31.15)
N	30	14	20	14	42	23	143
rs210446561 (*CEBPA*)	A	H	¾A	¾ H	½AH	LX	Global
C	25.00 (14.72 - 37.86)	10.71 (2.27 - 28.23)	12.50 (4.19 - 26.80)	14.29 (4.03 - 32.67)	10.71 (5.02 - 19.37)	8.70 (2.42 - 20.79)	13.99 (10.18 - 18.55)
G	75.00 (62.14 - 85.28)	89.29 (71.77 - 97.73)	87.50 (73.20 - 95.81)	85.71 (67.33 - 95.97)	89.29 (80.63 - 94.98)	91.30 (79.21 - 97.58)	86.01 (81.44 - 89.82)
N	23	16	16	14	61	23	153
rs207774429 (*RXRA*)	A	H	¾A	¾ H	½AH	LX	Global
C	71.74 (56.54 - 84.01)	65.63 (46.81 - 81.43)	65.63 (46.81 - 81.43)	64.29 (44.07 - 81.36)	68.85 (59.84 - 76.93)	69.57 (54.25 - 82.26)	68.30 (62.76 - 73.48)
T	28.26 (15.99 - 43.46)	34.38 (18.57 - 53.19)	34.38 (18.57 - 53.19)	35.71 (18.64 - 55.93)	31.15 (23.07 - 40.16)	30.43 (17.74 - 45.75)	31.70 (26.52 - 37.23)
N	44	26	30	24	95	41	260
rs133517803 (*RXRA*)	A	H	¾A	¾ H	½AH	LX	Global
A	32.56 (22.84 - 43.52)	5.77 (1.21 - 15.95)	33.33 (21.69 - 46.87)	20.83 (10.47 - 34.99)	23.40 (17.55 - 30.12)	45.00 (33.85 - 56.53)	27.43 (23.62 - 31.51)
G	67.44 (56.48 - 77.16)	94.23 (84.05 - 98.79)	66.67 (53.31 - 78.31)	79.17 (65.01 - 89.53)	76.60 (69.88 - 82.45)	55.00 (43.47 - 66.15)	72.57 (68.49 - 76.38)
rs136289117 (*RXRA*)	A	H	¾A	¾ H	½AH	LX	Global
C	1.16 (0.03 - 6.31)	1.92 (0.05 - 10.26)	0.00 (0.00 - 5.96)	0.00 (0.00 - 7.40)	0.00 (0.00 - 1.92)	0.00 (0.00 - 4.40)	0.39 (0.05 - 1.39)
T	98.84 (93.69 - 99.97)	98.08 (89.74 - 99.95)	100.00 (94.04 - 100.00)	100.00 (92.60 - 100.00)	100.00 (98.08 - 100.00)	100.00 (95.60 - 100.00)	99.61 (98.61 - 99.95)
rs209839910 (*RXRA*)	A	H	¾A	¾ H	½AH	LX	Global
C	97.67 (91.85 - 99.72)	98.08 (89.74 - 99.95)	100.00 (94.04 - 100.00)	95.83 (85.75 - 99.49)	98.31 (95.15 - 99.65)	96.15 (89.17 - 99.20)	97.81 (96.11 - 98.90)
T	2.33 (0.28 - 8.15)	1.92 (0.05 - 10.26)	0.00 (0.00 - 5.96)	4.17 (0.51 - 14.25)	1.69 (0.35 - 4.85)	3.85 (0.80 - 10.83)	2.19 (1.10 - 3.89)

**Table 3:** Unbiased expected heterozygosity (h_e_), observed heterozygosity (h_o_) and Hardy-Weinberg Equilibrium (HWE) p-values for SNPs in the *PPARG*, *CEBPA* and *RXRA* genes in an Argentinean crossbred population (Angus-Hereford-Limousin). A: purebred Angus; H. purebred Hereford; ¾A: 75 % Angus steers; ¾ H: 75 % Hereford steers; ½AH: 50 % Angus -50 % Hereford steers; LX: Limousine crossbred steers

SNP	Population											
	A			H			¾A			¾ H		
	h_e_	h_o_	HWE *p* value	h_e_	h_o_	HWE *p*-value	h_e_	h_o_	HWE *p*-value	h_e_	h_o_	HWE *p*-value
rs41610552 (*PPARG*)	0.44	0.50	0.49	0.30	0.36	0.56	0.36	0.33	0.63	0.22	0.25	1.00
rs207671117 (*PPARG*)	0.09	0.09	1.00	0.00	0.00	-	0.00	0.00	-	0.04	0.04	-
rs42016945 (*PPARG*)	0.44	0.41	0.73	0.34	0.42	0.55	0.44	0.30	0.10	0.31	0.29	1.00
rs210446561 (*CEBPA*)	0.38	0.50	0.15	0.20	0.21	1.00	0.22	0.25	1.00	0.25	0.29	1.00
rs207774429 (*RXRA*)	0.41	0.39	1.00	0.47	0.69	0.09	0.47	0.56	0.59	0.48	0.71	0.09
rs133517803 (*RXRA*)	0.44	0.37	0.31	0.11	0.12	1.00	0.45	0.67	0.01*	0.34	0.42	0.54
rs136289117 (*RXRA*)	0.02	0.02	-	0.04	0.04	-	-	-	-	-	-	-
rs209839910 (*RXRA*)	0.05	0.05	1.00	0.04	0.04	-	0.00	0.00	-	0.08	0.08	1.00
	½AH			LX						Global		
	h_e_	h_o_	HWE *p*-value	h_e_	h_o_	HWE *p*-value				h_e_	h_o_	HWE *p*-value
rs41610552 (*PPARG*)	0.36	0.42	0.14	0.49	0.55	0.51				0.38	0.42	0.72
rs207671117 (*PPARG*)	0.06	0.06	1.00	0.12	0.12	1.00				0.06	0.06	1.00
rs42016945 (*PPARG*)	0.37	0.32	0.17	0.45	0.29	0.03*				0.40	0.33	0.16
rs210446561 (*CEBPA*)	0.19	0.21	1.00	0.16	0.17	1.00				0.24	0.28	0.99
rs207774429 (*RXRA*)	0.43	0.59	<0.01*	0.43	0.44	1.00				0.43	0.56	<0.01*
rs133517803 (*RXRA*)	0.36	0.38	0.77	0.50	0.60	0.33				0.40	0.42	0.34
rs136289117 (*RXRA*)	-	-	-	-	-	-				0.01	0.01	1.00
rs209839910 (*RXRA*)	0.03	0.03	1.00	0.08	0.08	1.00				0.04	0.04	1.00

*Significant deviations from the theoretical proportions (*P* < 0.05)

SNP rs110793792, located in *CEBPA*, could not be genotyped by the Sequenom platform, reason why it was discarded from the analysis. The other SNP from this gene, rs210446561, was genotyped efficiently in only 143 samples. The analysis showed higher frequencies for allele C, with MAF ≥ 0.09 in the subpopulations (Table 2). The HWE test showed no deviations from the theoretical proportions. Values for h_e_ ranged from 0.16 (LX) to 0.38 (A), and h_o_ ranged from 0.17 (LX) to 0.50 (A) (Table 3).

Two of the SNPs from *RXRA*, rs207774429 and rs133517803, showed relatively balanced allele frequencies (MAF ≥ 0.06). It is worth mentioning that rs207774429, as happened with rs210446561 (*CEBPA*), was genotyped efficiently in only 153 samples. The other two SNPs, rs136289117 and rs209839910, showed very low variation and were not considered for association (Table 2). According to the HWE test, significant deviations were observed for rs207774429 in the whole population (*P* < 0.01) and rs133517803 in subpopulation ¾A (*P* < 0.01). The remaining SNPs showed no significant deviations from the theoretical proportions. Three of the SNPs in this gene were part of a linkage block constituted by three haplotypes, with two of them accounting for over 97 % of the haplotype frequencies (Additional file 4: Figure S1).

As we already mentioned, some of the SNPs selected for validation were not efficiently genotyped by Sequenom, but the rest showed different allele frequencies among the genetic groups. In general, the highest or lowest frequencies were observed in the LX subpopulation, which is historically and productively the most different breed, since Limousin is a European continental breed, and Angus and Hereford are Scottish. Deviations from the HWE proportions were observed mainly in crossbreeds, as expected. These deviations would also suggest the violation of some of Hardy-Weinberg assumptions and the existence of phenomena like selective mating, small population size, endogamy, and especially migration, considering the original purpose of the population, i.e., the evaluation of crossbreeding systems.

### Association study

When individual tests were performed, several genotype effects were observed on the evaluated traits. The least square means for each genotype, percentages of phenotypic variance explained by the SNPs, and substitution effects were estimated for all the evaluated traits and presented in Table 4.

**Table 4:** Association of SNPs in the *PPARG*, *CEBPA* and *RXRA* genes with meat quality traits in an Argentinean crossbred cattle population: least square means and standard deviation (s.d.) of the genotypic classes based on the individual polymorphisms, additive and dominance effects, substitution effect and percentage of phenotypic variance explained by the SNP (*σ*_*SNP*_^2^). N: number of samples; n.e.: non estimable; C18:0: stearic acid (%); C18:1 cis-9: oleic acid (%);C18:3 cis-6,9,12: ϒ-linolenic acid (%); C18:3 cis-9,12,15: α-linolenic acid (%); MUFA: monounsaturated fatty acids (%); Ω-6/Ω-3: omega-6/omega-3 proportion; BT: backfat thickness of beef (mm)

SNP/Trait	Least square means			Dominance effect	Aditive effect	Substitution effect	*σ*_*SNP*_^2^
rs207671117 (*PPARG*)	GG (*N* = 244)	GA (*N* = 16)	AA (*N* = 0)				
Ω-6/Ω-3	3.026 ± 0.081	2.314 ± 0.283	----	n.e.	0.712 ± 0.284 (*P* = 0.013) *	n.e.	0.40
rs41610552 (*PPARG*)	CC (*N* = 135)	CG (*N* = 109)	GG (*N* = 12)				
C18:1 cis-9	39.797 ± 0.276	40.445 ± 0.302	39.318 ± 0.737	0.888 ± 0.430 (*P* = 0.040)	0.240 ± 0.374 (*P* = 0.522)	-0.190 (C > G)	2.09
rs42016945 (*PPARG*)	CC (*N* = 146)	CT (*N* = 86)	TT (*N* = 27)				
C18:0	14.047 ± 0.199	13.586 ± 0.246	14.369 ± 0.414	-0.622 ± 0.295 (*P* = 0.036)	0.160 ± 0.218 (*P* = 0.462)	0.445 (C > T)	0.80
BT	3.641 ± 0.130	3.586 ± 0.160	3.064 ± 0.269	0.234 ± 0.195 (*P* = 0.231)	0.288 ± 0.143 (*P* = 0.045)	0.181 (C > T)	0.31
rs133517803 (*RXRA*)	GG (*N* = 132)	GA (*N* = 109)	AA (*N* = 16)				
C18:1 cis-9	40.115 ± 0.280	39.836 ± 0.286	41.650 ± 0.660	-1.047 ± 0.395 (*P* = 0.009)	0.768 ± 0.336 (*P* = 0.023)	1.241 (G > A)	1.77
18:3 cis-6,9,12	0.055 ± 0.006	0.045 ± 0.007	0.018 ± 0.017	0.013 ± 0.011 (*P* = 0.223)	0.018 ± 0.009 (*P* = 0.040)	0.012 (G > A)	0.35
C18:3 cis-9,12,15	0.796 ± 0.024	0.805 ± 0.025	0.644 ± 0.062	0.085 ± 0.039 (*P* = 0.030)	0.076 ± 0.032 (*P* = 0.019)	0.038 (G > A)	1.47
MUFA	48.044 ± 0.277	47.796 ± 0.283	49.690 ± 0.656	-1.071 ± 0.393 (*P* = 0.007)	0.823 ± 0.334 (*P* = 0.014)	1.306 (G > A)	1.35
BT	3.683 ± 0.141	3.481 ± 0.144	2.910 ± 0.353	0.185 ± 0.215 (*P* = 0.392)	0.387 ± 0.182 (*P* = 0.035)	0.303 (G > A)	0.95

* Calculated as the difference between the two genotypes detected in the population

Regarding SNPs in *PPARG*, rs207671117 showed a significant effect on Ω6/Ω3 (*P* < 0.05), but small genetic variation in general. An additive effect on BT was detected (*P* < 0.05) for rs42016945, while dominance effects were significant (*P* < 0.05) for rs41610552 on C18:1 cis-9 and rs42016945 on C18:0. For these SNPs, the percentages of phenotypic variance explained ranged from 0.31 to 2.09 %. SNP rs133517803 (*RXRA*) showed significant effects on several measures: additive effects were detected on C18:1 cis-9; C18:3 cis-6,9,12; C18:3 cis-9,12,15; MUFA and BT; while a dominance effect was detected on C18:1 cis-9; C18:3 cis-9,12,15 and MUFA. For these traits, the SNP explained from 0.35 to 1.77 % of the phenotypic variance (Table 4). No significant effects were observed on the other traits. It is worth mentioning that none of these effects reached the threshold for statistical significance in the FDR control by means of the Benjamini-Hochberg method considering 70 tests.

The explained percentages of variance were interestingly high for oleic acid and MUFA compared with other traits such as BT and stearic acid. In particular, the percentage of phenotypic variance explained by SNP rs133517803 in *RXRA* for oleic acid, and subsequently for MUFA, was high, which may suggest an important role of this gene in the oleic acid deposition in muscle.

If we consider the first approach, our results were partially consistent with previous reports from other authors. Sevane et al. [9] reported associations for SNP rs42016945 with several Ω-3 fatty acids. Here, rs42016945 showed a significant effect on fatty acid composition, but on C18:0 instead. We also found a possible effect on BT, which was interesting and expectable given the nature of PPARG. None of the mutations in *PPARG* reported in Chinese and Korean breeds [5, 7] was detected in our panel, probably due to different breed origins, although it is important to mention the finding of significant effects on BT in Chinese cattle, which is concordant with our findings in the European breeds. SNP rs110793792 (*CEBPA*) had been associated with BT and marbling in Chinese breeds [8], but these effects could not be tested in our population due to a genotyping problem. To our knowledge, there are no previous works reporting associations for *RXRA*, so this work may provide evidence that SNPs in this gene may be helpful for animal improvement by means of marker-assisted selection programs.

### Bioinformatic predictions

Since SNP rs207671117 was located in the 5’ UTR of PPARG, we analysed the possible effects on mRNA stability and putative RBP binding sites. Two highly similar structures were obtained running the UTR sequences of the alternative variants in The Mfold Web Server, but the structure for allele A (ΔG = -42.60 kcal/mol) seemed slightly more stable than that for allele G (ΔG = -42.20 kcal/mol). When RBP binding sites were analysed (threshold = 0.8), we found that this SNP was located one base away from a putative binding site for protein FUS (SCORE = 7.36), which is important in maintaining genomic integrity. These same studies were performed for rs42016945 and we found that the structures provided by Mfold were quite different despite the similarity of energy values. The structure for allele G (ΔG = -27.70 kcal/mol) seemed more stable than structure for allele A (ΔG = -26.90 kcal/mol). According to the analysis on RBPDB, no sites for RBP were identified promptly at the mutated site, but the SNP was immediately next to a NONO (non-POU domain-containing octamer-binding protein) binding site (SCORE = 8.95). NONO is a protein involved in numerous nuclear processes like unwinding, recombination, DNA binding and regulation of splicing.

SNPs rs133517803 (*RXRA)* was located in a possible alternative promoter region. Therefore, we searched for putative transcription factor binding sites through PhysBinder. According to this tool, rs133517803 was located over an ESRRB (estrogen-related receptor beta) binding site (threshold = 308), whose role is still not clear.

## Conclusions

*PPARG* and *CEBPA* showed low to moderate variability in our mixed sample panel. Variations in these genes, along with *RXRA*, may explain part of the phenotypic variation in fat content and composition of meat, especially SNPs in *RXRA*, which explained an important part of the variation in the highly heritable oleic acid percentage and MUFA. The molecular bases of the phenotypic differences may be partially explained by changes in RNA structures, RBP binding sites, codon usage frequencies and TF binding sites. The SNPs we analysed should be evaluated in independent populations with in-vitro and in-vivo analyses to explain the mechanisms by which these polymorphisms may be involved in the traits.

## Additional files

Additional file 1: Table S1. Genetic structure of the crossbred population used to perform validation and association studies. N: number of samples; A: purebred Angus; H: purebred Hereford; ¾A: 75 % Angus steers; ¾ H: 75 % Hereford steers; ½AH: 50 % Angus- 50 % Hereford steers; L: Limousin sire; LX: Limousin crossbred steers. (DOC 43 kb) Additional file 2: Table S2. Primers used to amplify and re-sequence the *PPARG* and *CEBPA* genes in a panel composed of 43 samples from nine cattle breeds with different meat quality. (DOC 42 kb) Additional file 3: Table S3. Fat content and composition in the local crossbred population (Angus-Hereford-Limousin). BT was measured in millimeters, IF was expressed as the amount of fat in 100 g of fresh muscle excluding the external adipose tissue, and the fatty acid content was expressed as percentage of total fatty acids. (DOC 43 kb) Additional file 4: Figure S1. Haplotypes (upper part) and linkage disequilibrium (lower part) among SNPs in the *PPARG* (A) and *RXRA* (B) genes in 260 samples from an Argentinean crossbred population (Angus-Hereford-Limousin, *N* = 260). Blocks were defined with the solid spine of LD method and indicated in thick lines. r2 values are indicated inside the boxes of the linkage scheme. PPARG5UTR represents SNP rs207671117. (TIF 512 kb)

## References

[1] Shahidi F, Kerry J, Kerry J, Ledward D (2002). Lipid-derived flavors in meat products. Meat processing: improving meat quality.

[2] Du M, Yin J, Zhu MJ (2010). Cellular signaling pathways regulating the initial stage of adipogenesis and marbling of skeletal muscle. Meat Sci.

[3] Hausman GJ, Dodson MV, Ajuwon K, Azain M, Barnes KM, Guan LL, Jiang Z, Poulos SP, Sainz RD, Smith S, Spurlock M, Novakofski J, Fernyhough ME, Bergen WG. Board-invited review: the biology and regulation of preadipocytes and adipocytes in meat animals. J Anim Sci. 2009;87(4):1218–46. 10.2527/jas.2008-1427 18849378

[4] Barendse W (2011). Haplotype Analysis Improved Evidence for Candidate Genes for Intramuscular Fat Percentage from a Genome Wide Association Study of Cattle. PLoS One.

[5] Fan YY, Zan LS, Fu CZ, Tian WQ, Wang HB, Liu YY, Xin YP. Three novel SNPs in the coding region of PPARγ gene and their associations with meat quality traits in cattle. Mol Biol Rep. 2011;38(1):131–7. 10.1007/s11033-010-0086-2 20306301

[6] He H, Liu X, Gu Y, Liu Y, Yang J (2011). Effect of genetic variation of CEBPA gene on body measurement and carcass traits of Qinchuan cattle. Mol Biol Rep.

[7] Oh D, Lee Y, Lee C, Chung E, Yeo J (2011). Association of bovine fatty acid composition with missense nucleotide polymorphism in exon 7 of peroxisome proliferator-activated receptor gamma gene. Anim Genet.

[8] Wang H, Zan LS, Wang HB, Song FB (2011). A novel SNP of the C/EBPα gene associated with superior meat quality in indigenous Chinese cattle. Gen Mol Res.

[9] Sevane N, Armstrong E, Cortés O, Wiener P, Pong Wong R, Dunner S, Gemqual Consortium. Association of bovine meat quality traits with genes included in the PPARG and PPARGC1A networks. Meat Sci. 2013;94:328–35. 10.1016/j.meatsci.2013.02.014 23567132

[10] USDA (United States Department of Agriculture). Livestock and Poultry: World Markets and Trade. Foreign Agricultural Service. 2015. http://apps.fas.usda.gov/psdonline/circulars/livestock_poultry.pdf. Accessed 18 March 2016.

[11] Giovambattista G, Ripoli MV, Lirón JP, Villegas Castagnasso EE, Peral-García P, Lojo MM (2001). DNA typing in a cattle stealing case. J Forensic Sci.

[12] Goszczynski DE, Mazzucco JP, Ripoli MV, Villarreal EL, Rogberg-Muñoz A, Mezzadra CA, Melucci LM, Giovambattista G. Characterisation of the bovine gene LIPE and possible influence on fatty acid composition of meat. Meta Gene. 2014;16(2):746–60. 10.1016/j.mgene.2014.09.001 PMC4287880 25606458

[13] Larkin MA, Blackshields G, Brown NP, Chenna R, McGettigan PA, McWilliam H, Valentin F, Wallace IM, Wilm A, Lopez R, Thompson JD, Gibson TJ, Higgins DG. Clustal W and Clustal X version 2.0. Bioinformatics. 2007;23:2947–8. 10.1093/bioinformatics/btm404 17846036

[14] The Single Nucleotide Polymorphism Database (dbSNP). http://www.ncbi.nlm.nih.gov/snp. Accessed 6 January 2016.

[15] Sequenom, Inc. https://www.sequenom.com. Accessed 6 January 2016.

[16] Barrett JC, Fry B, Maller J, Daly MJ (2005). Haploview: analysis and visualization of LD and haplotype maps. Bioinformatics.

[17] Rousset F (2008). GENEPOP’007: a complete re-implementation of the GENEPOP software for Windows and Linux. Mol Ecol Res.

[18] Schneider S, Roessli D, Excoffier L (2000). Arlequin, a software for Population Genetics Data Analysis.

[19] SAS software. Copyright, SAS Institute Inc., Cary, NC, USA.

[20] Falconer DS, Mackay TFC (1996). Introduction to Quantitative Genetics.

[21] Benjamini Y, Hochberg Y (1995). Controlling the false discovery rate: a practical and powerful approach to multiple testing. J R Statist Soc B.

[22] The Codon Usage Database. http://www.kazusa.or.jp/codon/. Bos taurus [gbmam]: 13374. Accessed 6 January 2016.

[23] Zuker M (2003). Mfold web server for nucleic acid folding and hybridization prediction. Nucleic Acids Res.

[24] Cook KB, Kazan H, Zuberi K, Morris Q, Hughes TR (2011). RBPDB: a database of RNA-binding specificities. Nucleic Acids Res.

[25] Broos S, Soete A, Hooghe B, Moran R, van Roy F, De Bleser P (2013). PhysBinder: Improving the prediction of transcription factor binding sites by flexible inclusion of biophysical properties. Nucleic Acids Res.

[26] Bovine HapMap Consortium (2009). Genome-wide survey of SNP variation uncovers the genetic structure of cattle breeds. Science.

