# Supplementary material for: Genetic characterisation of PPARG, CEBPA and RXRA, and their influence on meat quality traits in cattle
Source: J Anim Sci Technol. 2016 Apr 1;58:14. doi: 10.1186/s40781-016-0095-3 (PMC4818460; doi:10.1186/s40781-016-0095-3)
Supplement: Additional file 1: Table S1. — Genetic structure of the crossbred population used to perform validation and association studies. N: number of samples; A: purebred Angus; H: purebred Hereford; ¾A: 75 % Angus steers; ¾ H: 75 % Hereford steers; ½AH: 50 % Angus- 50 % Hereford steers; L: Limousin sire; LX: Limousin crossbred steers. (DOC 43 kb) [file 40781_2016_95_MOESM1_ESM.doc]

|  | | | | | |
| --- | --- | --- | --- | --- | --- |
| **Sire breed** | **Dam breed** | **Steer breed** | **Genetic group** | **N** |  |
| A | A | A | A | 44 |  |
| H | H | H | H | 26 |  |
| A | H | AH | ½AH | 95 |  |
| H | A | HA |  |
| AH | AH | AHAH |  |
| HA | HA | HAHA |  |
| A | AH | AAH | ¾A | 30 |  |
| A | HA | AHA |  |
| H | AH | HAH | ¾H | 24 |  |
| H | HA | HHA |  |
| L | AH | LAH | LX | 41 |  |
| L | HA | LHA |  |
|  |  |  |  | |  |
| **Slaughter group** | **Date of slaughter** | **N** | **Genetic groups** | |  |
| 1 | 27 October 2008 | 50 | A (6), H (3), ¾A (7), ¾H (6), ½AH (23), LX (5) | |  |
| 2 | 4 January 2011 | 55 | A (8), H (7), ¾A (5), ¾H (10), ½AH (19), LX (6) | |  |
| 3 | 22 September 2011 | 29 | A (3), ¾A (12), ¾H (4), ½AH (10) | |  |
| 4 | 18 October 2011 | 29 | A (9), H (3), ¾A (3), ¾H (3), ½AH (8), LX (3) | |  |
| 5 | 3 January 2012 | 30 | A (6), H (2), ¾A (2), ¾H (1), ½AH (8), LX (11) | |  |
| 6 | 7 August 2012 | 25 | A (6), H (3), ½AH (10), LX (6) | |  |
| 7 | 17 September 2012 | 28 | A (6), H (3), ¾A (1), ½AH (15), LX (3) | |  |
| 8 | 16 October 2012 | 14 | H (5), ½AH (2), LX (7) | |  |

**Genetic structure of the crossbred population**

**Table S1.** Genetic structure of the crossbred population used to perform validation and association studies. N: number of samples; A: purebred Angus; H: purebred Hereford; ¾A: 75% Angus steers; ¾H: 75% Hereford steers; ½AH: 50% Angus- 50% Hereford steers; L: Limousin sire; LX: Limousin crossbred steers.
