# Supplementary material for: Genetic characterisation of PPARG, CEBPA and RXRA, and their influence on meat quality traits in cattle
Source: J Anim Sci Technol. 2016 Apr 1;58:14. doi: 10.1186/s40781-016-0095-3 (PMC4818460; doi:10.1186/s40781-016-0095-3)
Supplement: Additional file 2: Table S2. — Primers used to amplify and re-sequence the PPARG and CEBPA genes in a panel composed of 43 samples from nine cattle breeds with different meat quality. (DOC 42 kb) [file 40781_2016_95_MOESM2_ESM.doc]

| **Gene** | **Sequence (5' - 3')** | **Size (bp)** | **Tann (ºC)** | **Region** |
| --- | --- | --- | --- | --- |
| PPARG | TCAACCAAGGGTGGGCAGA | 299 | 67.5 | Promoter |
|  | TGTGACAGCGAAGGGCTCA |  |  |  |
|  | GACGCAACAGCTTGTCACAGC | 471 | 63.0 | Promoter + Exon 1 |
|  | CGCAAGAGCAGCAAGTTAAGC |  |  |  |
|  | TCAGGGCTAACGTCACAGCTGGT | 364 | 63.0 | Exon 2 |
|  | CCACAGCCATTAGTCCTGGGAGC |  |  |  |
|  | TCCTGTGATGATTGTCTGCTCCTGT | 211 | 62.0 | Exon 3 |
|  | ACCTTGCATCCTTCACAAGCATGA |  |  |  |
|  | CCCTCGCCCATATTCCTTTGTAGGG | 199 | 62.0 | Exon 4 |
|  | GAGAAGCGTCGAGTGCGGGC |  |  |  |
|  | GCACTCATTCATCCTGCCCTTTCTC | 273 | 62.0 | Exon 5 |
|  | ACCCGGCCACCCCAAATGAA |  |  |  |
|  | CAGGAGCCCAGCAAAGAGGT | 304 | 62.0 | Exon 6 |
|  | CAGCAAACTCGAACTTGGGC |  |  |  |
|  | CAGGTTTGCTGAACGTGAAGC | 838 | 64.0 | Exon 7 |
|  | GCTGCTGCTGGTGAGTGAGA |  |  |  |
| CEBPA | GCAGGAGGTAGTAGGCGTTG | 492 | 61.0 | Promoter + Exon |
|  | GTCGATGGACGTCTCGTGTT |  |  |  |
|  | GCGGCAACGACTTTGACTAC | 360 | 61.0 | Exon |
|  | CCGGTACTCGTTGCTGTTCT |  |  |  |

**Primers used in PCR reactions**

**Table S2**. Primers used to amplify and re-sequence the *PPARG* and *CEBPA* genes in a panel composed of 43 samples from nine cattle breeds with different meat quality. Tann: temperature used in the PCR reaction.
