# Supplementary material for: Genetic characterisation of PPARG, CEBPA and RXRA, and their influence on meat quality traits in cattle
Source: J Anim Sci Technol. 2016 Apr 1;58:14. doi: 10.1186/s40781-016-0095-3 (PMC4818460; doi:10.1186/s40781-016-0095-3)
Supplement: Additional file 3: Table S3. — Fat content and composition in the local crossbred population (Angus-Hereford-Limousin). BT was measured in millimeters, IF was expressed as the amount of fat in 100 g of fresh muscle excluding the external adipose tissue, and the fatty acid content was expressed as percentage of total fatty acids. (DOC 43 kb) [file 40781_2016_95_MOESM3_ESM.doc]

| **Trait** | **N** | **Mean** | **SD** | **Min** | **Max** |
| --- | --- | --- | --- | --- | --- |
| Backfat thickness (BT) | 259 | 3.55 | 1.54 | 0.50 | 10.00 |
| Intramuscular fat (IF) | 260 | 9.65 | 3.71 | 3.36 | 26.20 |
| Myristic acid (C14:0) | 258 | 2.47 | 0.60 | 1.09 | 4.01 |
| Myristoleic acid (C14:1) | 258 | 0.52 | 0.25 | 0.12 | 1.50 |
| Palmitic acid (C16:0) | 258 | 27.07 | 2.28 | 22.32 | 31.93 |
| Palmitoleic acid (C16:1) | 258 | 4.22 | 0.80 | 2.36 | 7.46 |
| Stearic acid (C18:0) | 258 | 14.11 | 2.08 | 8.55 | 20.84 |
| Oleic acid (C18:1 cis-9) | 258 | 40.07 | 2.90 | 32.96 | 48.25 |
| Linoleic acid (C18:2 cis-9,12) | 258 | 3.10 | 1.38 | 1.04 | 7.56 |
| γ-linolenic acid (C18:3 cis-6,9,12) | 258 | 0.05 | 0.07 | 0.00 | 0.52 |
| α-linolenic acid (C18:3 cis-9,12,15) | 258 | 0.82 | 0.35 | 0.28 | 2.21 |
| Saturated fatty acid (SFA) | 258 | 45.16 | 3.06 | 38.67 | 52.8 |
| Monounsaturated fatty acid (MUFA) | 258 | 47.84 | 2.71 | 38.92 | 54.91 |
| Ω6/Ω3 proportion | 258 | 2.91 | 1.25 | 1.11 | 11.50 |

**Summary of measurements**

**Table S3**. Fat content and composition in the local crossbred population (Angus-Hereford-Limousin). BT was measured in millimeters, IF was expressed as the amount of fat in 100 g of fresh muscle excluding the external adipose tissue, and the fatty acid content was expressed as percentage of total fatty acids.
