# Supplementary figures and images for: Genetic characterisation of PPARG, CEBPA and RXRA, and their influence on meat quality traits in cattle
Source: J Anim Sci Technol. 2016 Apr 1;58:14. doi: 10.1186/s40781-016-0095-3 (PMC4818460; doi:10.1186/s40781-016-0095-3)

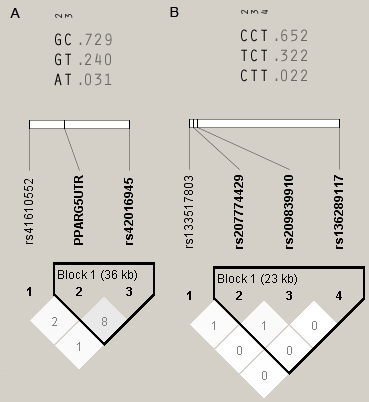

Supplement: Additional file 4: Figure S1. — Haplotypes (upper part) and linkage disequilibrium (lower part) among SNPs in the PPARG (A) and RXRA (B) genes in 260 samples from an Argentinean crossbred population (Angus-Hereford-Limousin, N = 260). Blocks were defined with the solid spine of LD method and indicated in thick lines. r2 values are indicated inside the boxes of the linkage scheme. PPARG5UTR represents SNP rs207671117. (TIF 512 kb) [file 40781_2016_95_MOESM4_ESM.tif]
